# Two-dimensional gel electrophoresis data for proteomic profiling of *Sporothrix* yeast cells

**DOI:** 10.1016/j.dib.2014.11.004

**Published:** 2014-12-01

**Authors:** Anderson Messias Rodrigues, Paula H. Kubitschek-Barreira, Geisa Ferreira Fernandes, Sandro Rogério de Almeida, Leila M. Lopes-Bezerra, Zoilo Pires de Camargo

**Affiliations:** a Department of Microbiology, Immunology and Parasitology, Cell Biology Division, Federal University of São Paulo (UNIFESP), São Paulo, Brazil; b Department of Cellular Biology, The Roberto Alcantara Gomes Institute of Biology, University of Estado Rio de Janeiro (UERJ), Brazil; c Department of Clinical and Toxicological Analysis, Faculty of Pharmaceutical Sciences, University of São Paulo (USP), São Paulo, Brazil

## Abstract

Sporotrichosis is a chronic infection of the skin and subcutaneous tissues of human and other mammals caused by a complex of cryptic dimorphic fungi in the plant-associated order Ophiostomatales. With major differences between routes of transmission, *Sporothrix* infections are emerging as new threat in tropical and subtropical areas, particularly in form of outbreaks. The mechanisms underlying the pathogenesis and invasion of *Sporothrix* spp. are still poorly understood and many virulence factors remain unidentified. In this scenario, a global analysis of proteins expressed by clinical *Sporothrix* species combined with the identification of seroreactive proteins is overdue. Optimization of sample preparation and electrophoresis conditions are key steps toward reproducibility of gel-based proteomics assays. We provide the data generated using an efficient protocol of protein extraction for rapid and large-scale proteome analysis using two-dimensional gel electrophoresis. The protocol was established and optimized for pathogenic and non-pathogenic *Sporothrix* spp. including *Sporothrix brasiliensis* (CBS 132990), *Sporothrix schenckii sensu stricto* (CBS 132974), *Sporothrix globosa* (CBS 132922), and *Sporothrix mexicana* (CBS 120341). The data, supplied in this article, are related to the research article entitled “Immunoproteomic analysis reveals a convergent humoral response signature in the *Sporothrix schenckii* complex” (Rodrigues et al., 2014 [1]).

**Table:** 

**Specifications table**	

Subject area	*Biology*

More specific subject area	*Medical Mycology, Immunology*
Type of data	*Image*
How data was acquired	*Scanning electron microscopy FEI Quanta^™^ FEG 250, Ettan IPGphor 3 Isoelectric Focusing Unit, Image Scanner III*
Data format	*Analyzed*
Experimental factors	*Sporothrix yeast cells from clinical and environmental species were grown in Brain Heart Infusion medium for 7 days at 36 °C and subjected to a whole cell protein extraction*
Experimental features	*All the samples were separated using two-dimensional gel electrophoresis*
Data source location	*São Paulo, Brazil*
Data accessibility	*Within this article *


**Value of the data**	

• Morphological and morphometric analyses using scanning electron microscopy revealed distinct cellular architecture of *Sporothrix* species.	
• A gel-based proteomic profiling of pathogenic and non-pathogenic *Sporothrix* spp. is proposed.	
• Data from an efficient method of protein extraction with application to 2D gel electrophoresis and mass spectrometry analyses.	
• The optimized protocol is useful for comparative proteome analysis of *Sporothrix* spp., including immunoblotting and 2D-DIGE.	

## 1 Data, experimental design, materials and methods

### 1.1 *Sporothrix* culture conditions and morphological characterization

*Sporothrix brasiliensis* (CBS 132990), *Sporothrix schenckii sensu stricto* (CBS 132974), *Sporothrix globosa* (CBS 132922), and *Sporothrix mexicana* (CBS 120341) were maintained in Sabouraud dextrose agar slants (Difco, Detroit, USA) and cultivated for 10–14 days at 25 °C prior to use. These isolates were chosen based on geographical origin and genetic diversity [2–5], virulence profile [6,7], and physiological response to antifungal agents [8,9]. Approximately 2×10^6^ conidia (viable cells 90%) were used to inoculate 500 ml flasks containing 100 ml of Brain Heart Infusion Broth (Difco, Detroit, USA). The cultures were incubated at 36 °C in triplicate in a rotary shaker (Multitron II – Infors HT, Switzerland) with constant orbital agitation (110 rpm) for 7 days.

**Fig. 1:**
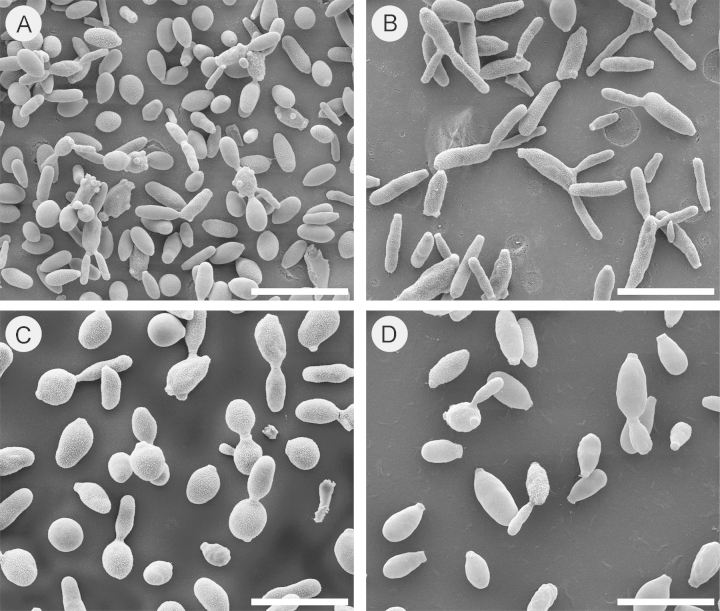
SEM-image of the parasitic phase of pathogenic and non-pathogenic *Sporothrix* species. The species embedded in the clinical clade are represented by reference clinical strains collected during the largest epidemic of sporotrichosis in South America. (A) Multi-budding yeasts of *S. brasiliensis* (CBS 132990=Ss54) clade I; (B) classical cigar-shaped yeasts of *S. schenckii* (CBS 132974=Ss118) clade II; (C) *S. globosa* (CBS 132922=Ss06) clade III. The ancestral non-virulent *S. mexicana* (CBS 120341) clade IV presented irregular budding yeasts. Bar=10 µm.

The morphological characterization of *Sporothrix* spp. was performed by scanning electron microscopy (SEM), especially because we were comparing species with different ecological and pathogenic behavior. *Sporothrix* yeast cells, obtained as described above, were harvested by centrifugation and washed twice in phosphate buffered saline (PBS). The cells were fixed overnight in 2.5% glutaraldehyde containing 0.1 M sodium cacodylate buffer (pH 7.2) washed in the same buffer and adhered onto poly-l-lysine-coated coverslips. Cells were post-fixed in 1% OsO_4_ containing 0.1 M sodium cacodylate, followed by 1% tannic acid for 30 min, each with appropriate washes. After osmium/sodium cacodylate, samples were immersed in 1% OsO_4_, washed in ultra-pure water, dehydrated in an ethanol series dilution (50%, 70%, 90%, and 100%), and critical-point dried with CO_2_ (Balzers CPD 030). Specimens were ion-sputtered with a 25-nm gold layer using a Leica EM SCD500 to avoid a charge effect while searching for a suitable site. SEM images were obtained using a FEI Quanta^™^ FEG 250 (Fei Company, USA) at a 5 kV accelerating tension (Federal University of São Paulo, Electron Microscopy Facility [CEME]) [1]. A representative image of *Sporothrix* yeast cells is shown in Fig. 1.

### 1.2 Morphological characteristics of *Sporothrix* yeast cells

**Fig. 2:**
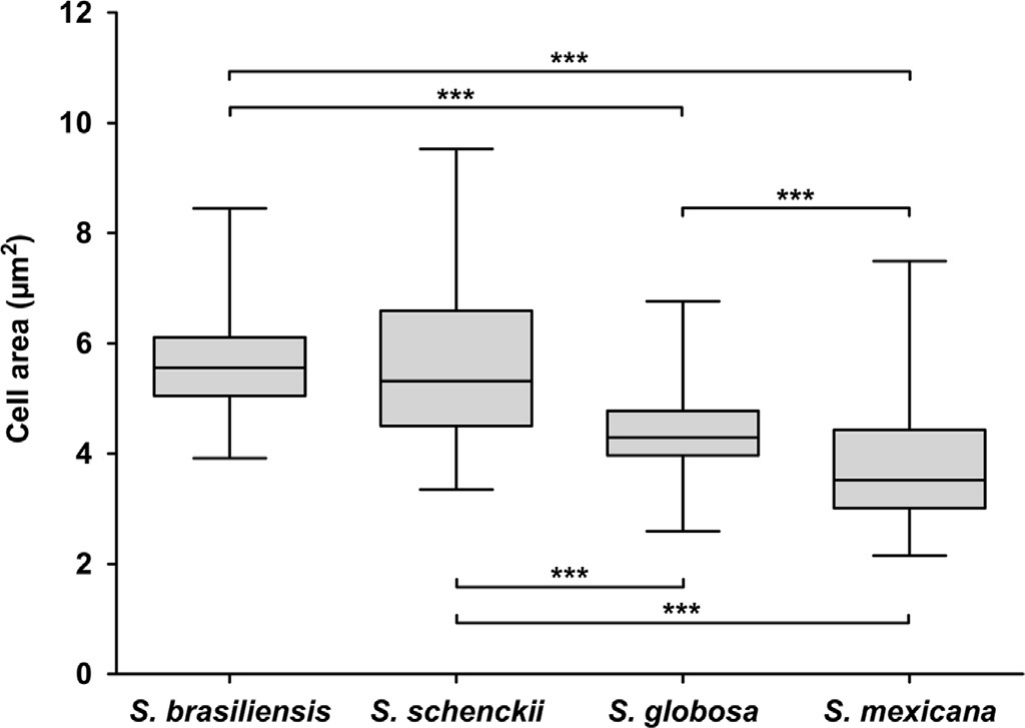
Morphometric characteristics of *Sporothrix brasiliensis* (CBS 132990), *S. schenckii s. str.* (CBS 132974), *S. globosa* (CBS 132922), and *S. mexicana* (CBS 120341) yeast cells. ^⁎⁎⁎^*p*<0.0001 in one-way ANOVA followed by Tukey׳s tests. The lines in the boxes indicate medians and the whiskers indicate maximum and minimum values.

*Sporothrix* yeast cells were processed and analyzed using Image J 1.44 C morphometric software (NIH, Bethesda, Maryland, USA; http://rsb.info.nih.gov/ij/). All measurements were estimated on the basis of the results obtained with at least 100 yeast cells/isolate from a 7-day-old culture on Brain Heart Infusion Broth at 36 °C as described above. Data were analyzed using one-way analysis of variance (ANOVA) with Tukey׳s multiple comparison post-hoc test. A *p*-value <0.05 was considered to indicate significant differences, and analyses were performed using GraphPad (GraphPad Prism v. 5.00 for Windows, San Diego, California, USA, www.graphpad.com). A graphic showing the variation in cell area is provided below (Table 1; Fig. 2).

### 1.3 Protein extraction method

**Fig. 3:**
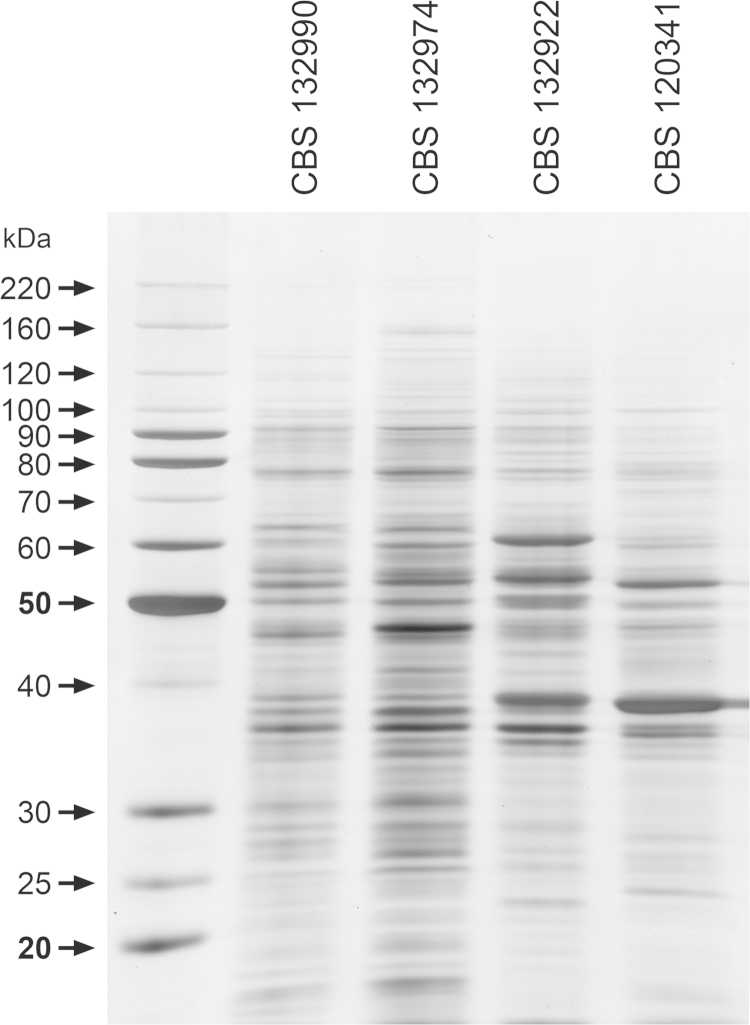
Protein profile from *Sporothrix* spp. after 7 days of incubation at 36 °C, 110 rpm in Brain Heart Infusion (BHI) media. The cell extracts were subjected to 1D SDS-PAGE and the proteins developed by silver staining. The molecular masses (in kDa) of standard proteins are given to the left of the gel (BenchMarkTM Protein Ladder, Invitrogen). CBS 132990=*S. brasiliensis*; CBS 132974=*S. schenckii*; CBS 132922=*S. globosa*; CBS 120341=*S. mexicana*.

Yeast cells obtained as described above were collected by centrifugation at 5000*g* for 10 min (4 °C) and then washed three times in ultrapure water. The final pellet (around 5 mL) was frozen in liquid nitrogen and disrupted by grinding with a pestle until a fine powder was obtained. The powder was suspended in 4 ml of Tris-Ca^2+^ buffer (20 mM Tris–HCl pH 8.8, 2 mM CaCl_2_) [10] containing a commercial cocktail of protease inhibitors (1:100) (GE Healthcare, USA), RNAse, and DNAse enzymes (1:100) (GE Healthcare, USA); and then glass beads (Sigma 425–600 µm) were added and the mixture vigorously vortexed for 30 min at 4 °C. Cell debris and glass beads were removed by centrifugation (11,000*g*, 4 °C, 10 min) and dithiothreitol (20 mM) added to the supernatant as described elsewhere [11]. Protein concentrations were determined by the Bradford method [12] and the cell extracts kept at −80 °C until use. Proteins extracts were evaluated according to (i) the amount of protein extracted; (ii) the diversity of bands; (iii) the integrity of samples as well as the reproducibility of extraction. Approximately 2 µg of protein preparations were resolved by 1D SDS-PAGE, yielding molecules ranging in size from 270 to 10 kDa with clear differences in protein profiles. The Tris–Ca^2+^ extraction protocol was suitable for the study of *Sporothrix* antigenic molecules, generating samples with a high amount of protein with no degradation. A representative image of *Sporothrix* proteins resolved by 1D SDS-PAGE is shown in Fig. 3.

### 1.4 Two-dimensional gel electrophoresis.

**Fig. 4:**
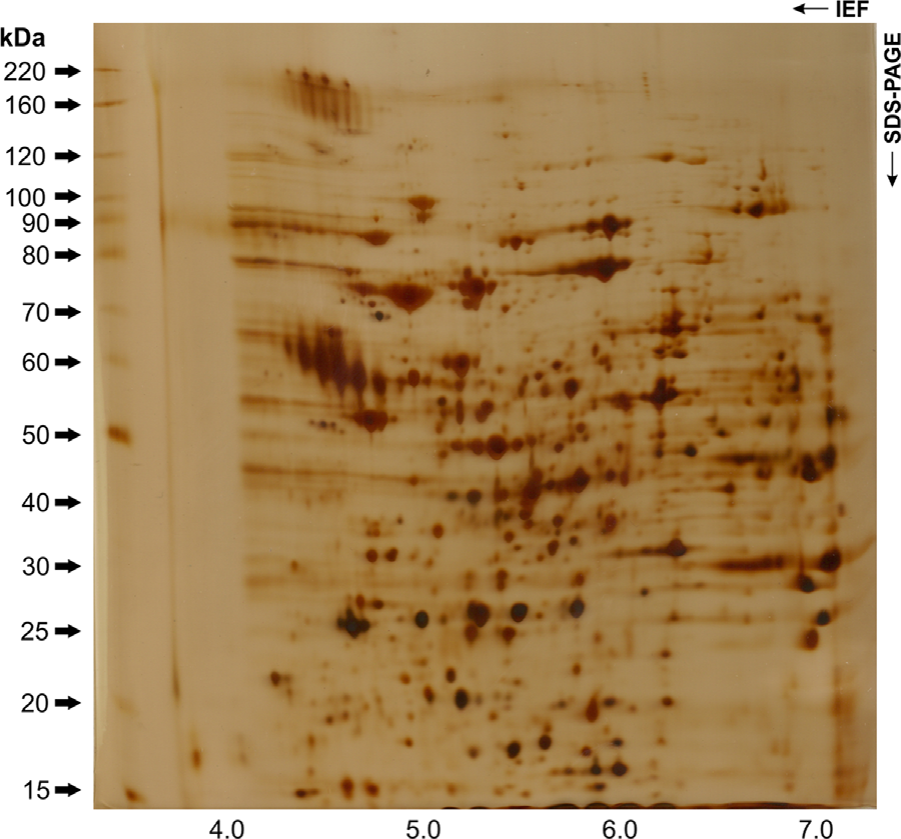
Optimization of protein extraction and 2D gel electrophoresis revealed a complex proteomic profiling for *Sporothrix schenckii* (CBS 132974). The separation of yeast proteins was performed on 13 cm strips over isoelectric point (p*I*) gradient of 4–7. Gel was loaded with 300 μg of total protein and silver stained.

To assess the complexity of the samples, 300 µg of protein was fractionated by 2D electrophoresis. Proteins were successfully precipitated using the 2D clean-up kit (GE Healthcare, Piscataway, NJ, USA) following the manufacturer׳s recommendations. Initially, for reproducibility, immobilized pH gradient (IPG) strips ranging from 3 to 10 were employed. The resulting gels had low resolution with a concentration of spots in the pH range of 4–7. Thus, the experiments were conducted using an immobilized pH gradient of 4–7, which provided better sample separation and improved resolution. Proteins were diluted with rehydration solution (7 M urea, 2 M thiourea, 2% CHAPS, 1.2% DeStreak, 2% vol/vol isoelectric focusing [IEF] buffer pH 4–7, and trace bromophenol blue) to a final volume of 250 µl. IEF was performed using a Ettan IPGphor III system (GE Healthcare, USA). IPG strips (pH 4–7, 13 cm) were rehydrated at 30 V for 12 h. Proteins were subsequently focused at 200 V for 2 h, 500 V for 2 h, 1000 V for 5 h, and then a gradient applied from 1000 to 5000 V for 2 h. Finally, the voltage was set to 5000 V until 60,000 Vh. All IEF experiments were performed at 20 °C. After one-dimensional IEF, the IPG strips were reduced for 15 min with 1.5% dithioerythritol and alkylated for 15 min with 2.5% iodocetamide in equilibration buffer (6 M urea, 50 mM Tris–HCl pH 6.8, 30% glycerol, and 2% SDS). Equilibrated strips were placed on homogeneous 10% polyacrylamide gels and sealed with 0.5% low-melting-point agarose and separated at 10 °C using a Hoefer SE 600 unit (15 mA/gel for 30 min and then 23 mA/gel until the dye front reached the bottom of the gel). Proteins were developed with silver staining [13]. Proteome maps were recorded using the Image Scanner III (GE Healthcare, USA). A representative image of *Sporothrix schenckii* proteins resolved by 2D gel electrophoresis is shown in Fig. 4. In summary, the protocol used for protein extraction is compatible with immunoblotting (see Fig. 2 in Ref. [1]), 2D-DIGE (see Fig. 3 in Ref. [1]) and mass spectrometry (see Fig. 4 in Ref. [1]), demonstrating the potential for the discovery of new fungal antigens.

## Figures and Tables

**Table 1:** Morphological and morphometric characteristics of *Sporothrix* yeast cells.

**Species**	**Isolate**	**Size**^a^			**Morphology**
		**Area (µm**^**2**^**)**	**Length (µm)**	**Width (µm)**	
*S. brasiliensis*	CBS 132990	5.62±0.87	3.47±0.46	1.91±0.30	Ellipsoidal
*S. schenckii*	CBS 132974	5.52±1.29	4.63±0.80	1.2±0.25	Cigar-shaped
*S. globosa*	CBS 132922	4.35±0.72	2.53±0.36	2.07±0.30	Globose
*S. mexicana*	CBS 120341	3.77±1.03	2.99±0.54	1.56±0.25	Narrow ellipsoidal

a Median±standard deviation.
